# Men’s Intentions to Recommend Professional Help-Seeking to Their Partners in the Postpartum Period: the Direct and Indirect Effects of Gender-Role Conflict

**DOI:** 10.3390/ijerph16204002

**Published:** 2019-10-19

**Authors:** Catarina Luís, Maria Cristina Canavarro, Ana Fonseca

**Affiliations:** 1 Faculty of Psychology and Educational Sciences, University of Coimbra; Coimbra, 3000-115 Coimbra, Portugal; 2 Center for Research in Neuropsychology and Cognitive-Behavioral Intervention, Faculty of Psychology and Educational Sciences, University of Coimbra, Coimbra, 3000-115 Coimbra, Portugal

**Keywords:** experiential avoidance, gender-role conflict, intention to seek professional help, men’s intentions to recommend professional help-seeking, postpartum mood and anxiety disorders, stigma

## Abstract

Women’s partners may act as facilitators of professional help-seeking for mental health problems in the postpartum period. This study aimed to examine the sociodemographic and clinical correlates of men’s intentions to recommend professional help-seeking to their partners if they display postpartum mood and anxiety disorders and to explore the relationship between gender-role conflict and the intention to recommend help-seeking. A cross-sectional study included 214 adult men in a heterosexual relationship with a partner within the reproductive age. Men presented a high intention to recommend professional help to their partners. All dimensions of gender-role conflict were directly associated with the intention to recommend professional help-seeking (*p* < 0.05). High levels of gender-role conflict (dimensions success, power and competition, and restricted emotionality) were found to lead to increased levels of stigma and lower levels of intention to seek professional help, which, in turn, translated into lower intention to recommend help-seeking. These results emphasize the importance of developing universal awareness-raising and education campaigns directed at men aiming to reduce levels of gender-role conflict and stigma, and normalize the use of mental health services, to increase men’s intentions to recommend professional help-seeking to their partners.

## 1. Introduction

Mood and anxiety disorders are prevalent conditions in the perinatal period. In most cases (64%), the clinical symptoms of anxiety experienced during pregnancy continue to occur into the postpartum period [1,2]. With regards to depression, its prevalence tends to increase from the first to the third trimester of pregnancy, affecting as many as 12% of women towards the end of their pregnancies [3] and affecting 13–20% of new mothers [4].

Despite its high prevalence and the availability of effective treatments for perinatal mood and anxiety disorders (PMAD) [5,6,7,8], only a small percentage of women seek professional help [9,10,11]. In fact, a study conducted within the Portuguese population found that only 13.6% of Portuguese women with clinically relevant depressive symptoms in the perinatal period seek professional help [10]. These results have been the subject of attention and concern, since not treating this symptomatology can lead to harmful effects not only for the woman herself [12], but for the mother–child interaction [13,14], the child’s development [15,16,17,18,19], and the broader family system [20].

In order to prevent these pervasive consequences, it is necessary to promote professional help-seeking amongst women. Women in the perinatal period prefer to resort to informal sources of help (e.g., social network) rather than formal sources of help (e.g., health professionals) to address their PMAD [11,21,22]. Moreover, there is some evidence that a woman’s support network may have a facilitating role in the professional help-seeking process [11,23], and that the decision to seek professional help is generally not made individually [24].

Being a significant element in a woman’s support network, partners play a key role in the professional help-seeking process [11,23]. They are one of the main persons with whom women share and discuss their emotional experiences in the perinatal period [10,11,24]. This privileged position allows partners to identify changes in a woman’s mental health and help her recognize the symptoms, since they are often not detected by the women themselves and may not be as visible to other elements of their social network [24,25]. Additionally, women’s partners often manifest concern with these symptoms during the perinatal period [11] and, therefore, they frequently play a role in the woman’s process of deciding to seek professional help and her acceptance of that decision [24]. The perception of encouragement from a partner has been found to lead to a greater intention to seek out professional help to address PMAD [21] and seems to result in an actual behavior of seeking professional help [26].

### 1.1. Men’s Intentions to Recommend Professional Help-seeking to their Partner with Postpartum Mood and Anxiety Disorders: Gender-Role Conflict

To the best of our knowledge, studies focused on the process of recommending professional help-seeking to others to address mental health problems are scarce. Recommending help-seeking to others is intimately related to the individual’s intention to seek help [27]. Existing studies with the general population suggest that most people would recommend professional help-seeking for mental health problems [28], including for PMAD [29]. However, there is some evidence of gender differences in the pattern of seeking and recommending help to others. Specifically, men are not only less likely to seek mental health services to cope with their emotional problems [30,31,32,33,34], but also report a lower intention to recommend professional help-seeking for postpartum depression when compared with women [29]. Additionally, it has been consistently demonstrated that, in comparison with women, men display more negative attitudes towards seeking help for mental health problems [30,34,35,36].

One way of trying to understand these gender differences is to consider them products of the socialization of the male gender role. The research on this topic began in the late 1980’s [37], gaining particular emphasis with the publication of Addis and Mahalik in 2003 [38]. The process of the socialization of gender roles has underpinned the learning of norms and stereotypes imposed by those around the individual, which indicate the underlying meaning of being a man and being a woman [39]. Thus, in different degrees and from a very early age, some men develop a *fear of femininity* (i.e., fear of demonstrating behaviors that are connoted as feminine), thus preventing them from acting in certain ways, or causing them suffering when performing certain actions, which results in *gender-role conflict* [40,41]. Gender-role conflict may be defined as a "psychological state in which socialised gender roles have negative consequences on the person or others" [42] (pp. 165–166), and can lead to the restriction of the human potential of those who experience it, and those surrounding the individual, interfering at a personal, professional, familial, and health level [40]. According to O’Neil [40], there are four patterns of gender-role conflict: (1) Concerns about success, power, and competition, exemplifying men who try to protect themselves from feelings of inferiority and weakness by trying to be better than others (success, power, and competition); (2) Difficulties and fears related to the expression of emotions and affections, as well as to the inability to express them appropriately (restrictive emotionality); (3) Difficulty in expressing feelings towards other men (restrictive affectionate behavior between men—homophobia); and (4) Difficulties in the balance between a family life and a career, which can result in stress, health problems, or overwork (conflict between work and family relations).

When considering professional help-seeking behaviors, research shows that men who experience high levels of gender-role conflict exhibit lower intentions to seek professional help [34] and more negative attitudes towards seeking help for psychological problems [31,43,44,45,46]. For these men, expressing feelings, exhibiting vulnerabilities, and having the feeling of losing control can be aspects that violate their idea of masculinity, thus threatening their identity [40]. Moreover, the act of seeking help from mental health services is associated with a perception of risks, such as rejection by their social network, the vision of oneself as a deviant member of society, and the sacrifice of autonomy [38]. To the extent of our knowledge, only one study examined the relationship between gender-role conflict and men’s intentions to recommend professional help-seeking for mental health problems, showing that higher levels of restrictive emotionality (a pattern of gender-role conflict) were associated with a lower intention to recommend help-seeking to others [47].

Despite the absence of studies directly exploring the relationship between gender-role conflict and men’s intentions to recommend professional help-seeking to their partners with PMAD, some preliminary evidence can be found. A qualitative study addressing the experiences of couples where women had postpartum depression revealed that men often fear making public the most intimate aspects of their family, believing that they should be able to solve problems on their own. Some of these men expressed that resorting to professional help to deal with the symptomatology presented by their partners increased their feelings of failure as a spouse [48]. Another qualitative study on the same theme found that some men try to maintain control of the situation through their avoidance, which translates into the nonexpression of concerns regarding the depressive symptomatology presented by their partner, or in a greater dedication to professional activity (e.g., less time spent at home) [49]. The role of gender-role conflict on men’s intentions to recommend professional help-seeking to their partner with PMAD should be further explored.

### 1.2. The Relationship between Gender-Role Conflict and Men’s Intentions to Seek and Recommend Professional Help: The Mediating Role of Experiential Avoidance and Stigma

As a self-regulatory strategy, experiential avoidance may play a role in explaining the relationship between gender-role conflict and the intention to seek and/or recommend professional help. Experiential avoidance is conceptualized as an individual’s tendency to avoid or control the frequency and form of internal experiences, especially undesirable ones (such as thoughts, emotions, memories, or sensations), as well as to avoid contexts that trigger them [50]. This tendency may guide the individual’s behavior and interfere with their interaction with situations and experiences they might value [51], since acting in accordance with their values often requires contact with these experiences [52].

Experiential avoidance can constitute a maladaptive response to gender-role conflict. As a result of the socialization of the male gender role, some boys are exposed to messages that dictate that men should not express negative emotions (e.g., sadness, fear), often being humiliated if they do so [53]. Moreover, it is possible that the attempt to control negative experiences, resulting from higher levels of experiential avoidance, may interfere with an individual’s personal values and the pursuit of their objectives [50], such as seeking help or recommending help-seeking to others. This hypothesis should be better explored.

Another variable that can play a mediating role in the relationship between gender-role conflict and the intention to seek and recommend professional help to their partners in the postpartum period is the stigma related to professional help-seeking. This type of stigma reflects the perception that those who seek and receive treatment for mental health problems are neither desired nor socially accepted [54] and may lead an individual into a state of discomfort or concern regarding what their significant others may wonder if they knew they were seeking or receiving professional help to cope with psychological problems [55]. Often, as a consequence of high levels of stigma, individuals do not seek treatment [56,57,58,59,60].

Although high levels of stigma towards professional help-seeking exist in both males and females [35,61,62], men generally perceive higher levels of this stigma [54,62]. This prominence of the stigma of professional help-seeking can be explained by male gender-role that emphasizes independence, self-sufficiency, and control. Within this perspective, seeking help may be perceived as an inability to resolve problems autonomously [38], as could recommending help-seeking to others, particularly to their partners in the postpartum period. Specifically, research shows that a greater conformity to the norms of the male gender role seems to be associated with higher levels of stigma of help-seeking, which in turn is associated with more negative attitudes towards help-seeking [63]. In addition, another study conducted with male university students found that higher levels of gender-role conflict were associated with higher levels of stigma, which were associated with more negative attitudes towards help-seeking, which in turn were associated with a lower intention to seek professional help [34]. As for men’s intentions to recommend professional help-seeking to others, a study found that gender-role conflict could directly and indirectly influence their intentions through stigma [47]. The role of stigma in the help-seeking behavior (to seek and recommend help to their partners) during the perinatal period should be further explored.

### 1.3. The Present Study 

For women, their partners are the main source of support and encouragement in the process of seeking professional help for PMAD. The present study focuses on men who are currently in a relationship with a woman within the reproductive age, and intends to characterize men’s intentions to recommend professional help-seeking to their partners if they display PMAD, as well as to understand the factors and mechanisms that can determine this behavioral intention. The focus on men’s behavioral intentions is relevant, since behavioral intentions are good predictors of behaviors [64,65], allowing us, therefore, to predict the behaviors of men in cases where their partners experience PMAD. 

**Figure 1:**
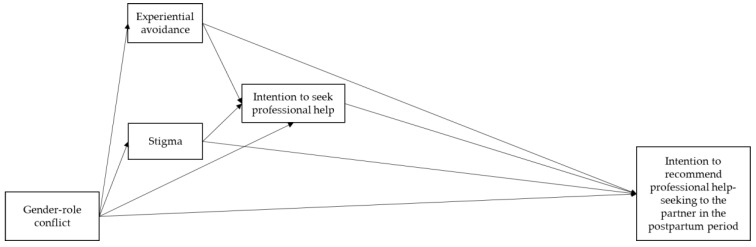
Conceptual scheme of the direct and indirect effects of gender-role conflict on the intention to recommend professional help-seeking to a partner with PMAD, through experiential avoidance, stigma, and the intention to seek professional help.

Specifically, the goals of this study are: (1) to analyze the sociodemographic and clinical correlates of men’s intentions to recommend professional help-seeking to their partners if they display PMAD; (2) to investigate the direct and indirect effects (through experiential avoidance, stigma, and the intention to seek professional help) of the relationship between the gender-role conflict and the intention to recommend professional help-seeking to their partners in the postpartum period (Figure 1).

## 2. Materials and Methods 

### 2.1. Procedure and Participants 

This cross-sectional study was approved by the Ethics Committee of the Faculty of Psychology and Educational Sciences, University of Coimbra. Inclusion criteria to participate in the study were: (1) Being male; (2) Being 18 years old or older; (3) Currently being in a heterosexual relationship (not/in cohabitation); (4) Being with a partner within the reproductive age (18–40 years old); (5) Being of Portuguese nationality and residing in Portugal; and (6) Having a level of understanding of the Portuguese language that allows the completion of the evaluation protocol.

The sample was collected in person and online, between December 2018 and April 2019. The online recruitment was conducted through advertisements on social networking websites, through general posts, posts particularly directed at the target population of the study, and posts in groups related to the research themes. A link to the Limesurvey^®^ online survey platform, with information regarding the project (researchers responsible for the study, objectives, voluntary nature of participation, and assurance of confidentiality and anonymity of the data obtained), was made available to the potential participants. Participants gave their informed consent through an affirmative answer to the question "Do you agree to participate in this study?" Only participants who gave their consent to participate in the study had access to the assessment protocol.

Regarding in-person recruitment, we contacted the Centro Infantil of Miranda do Corvo (Fundação Assistência, Desenvolvimento e Formação Profissional), the Creche da Santa Casa da Misericórdia of Penela, and the Creche e Jardim de Infância “O Caracol” of the hospital campus of the Hospitais da Universidade de Coimbra. After obtaining authorizations from the Directors of these institutions, male parents of the children attending these institutions were requested to participate, through the children’s educators as intermediaries. The potential participants were provided with the same aforementioned information about the project, and only after receiving a signed document of informed consent were they given access to the assessment protocol. Additionally, they were requested to return the duly filled evaluation protocol to their children’s educators within two weeks. A total of 38 participants (13.4%), out 284 potential participants returned the filled protocol. Additionally, the researchers used their own social networks to attempt to recruit more participants, as well as requesting those who had consented to participate in the study to reach out to their own acquaintances.

The final sample consisted of 214 men, in a relationship with a partner within the reproductive age. Of this, 74.8% (*n* = 160) of the sample was recruited online and 25.2% (*n* = 54) in person. The main characteristics of the sample are presented in Table 1. As set out in Table 1, the participants had a mean age of 32.61 years, and the majority of the sample was married or cohabiting, with housing in urban areas and a mean monthly income situated between 1001€ and 2000€. About 37.4% of the sample was experiencing the perinatal period with their partners (i.e., their partner was currently pregnant or had children under one year old), while 33.6% had older children (over a year old) and 29% did not have children.

### 2.2. Measures

Participants answered a sociodemographic and clinical form, including sociodemographic data (age, education, monthly income, area of residence, relationship status, parental status, and professional status), clinical data (prior history of psychopathology, prior history of psychological or psychiatric treatment, and current presence of psychopathology), and partner’s data (current pregnancy, current or prior history of psychopathology).

The Gender-Role Conflict Scale (GRCS) [66,67] was used to evaluate the conflicts of men with their gender-roles and consists of 37 items, with a Likert scale-type response format of 6 points, of 1 (Strongly disagree) to 6 (Strongly agree). It consists of four subscales: (1) Success, power, and competition (13 items; e.g., "I like to feel superior to other people"); (2) Restrictive emotionality (10 items; e.g., "I have difficulty telling others I care about them"); (3) Conflicts between work and family relations (six items; e.g., "My career, job or school affects the quality of my leisure or family life"); and (4) Restrictive affectionate behavior between men—homophobia (eight items; e.g., "Affection with other men makes me tense"). The first three subscales were used in the present study. The score of each subscale was calculated using the means of the score obtained in the items that compose it, and higher scores indicated a higher level of gender-role conflict. In the present study, Cronbach’s alpha values varied between 0.87 (Success, power, and competition dimension) and 0.90 (Restrictive emotionality and Conflicts between work and family relations dimensions).

The Acceptance and Action Questionnaire-II (AAQ-II) [68,69] was used to evaluate experiential avoidance (e.g., "My experiences and painful memories make it difficult for me to live a life that I value"). The AAQ-II is a unidimensional self-report scale consisting of seven items, with a 7-point Likert-type response format of 1 (Never true) to 7 (Always true). The total score was obtained through the sum of the items, and higher scores indicated greater experiential avoidance. In our sample, the Cronbach’s alpha was 0.91. 

In order to evaluate stigma, we used the Inventory of Attitudes Toward Seeking Mental Health Services (IATSMHS) [55,70]. This inventory consists of 24 items, answered on a 5-point Likert scale (0—I disagree to 4—I agree) and distributed into three subscales: (1) Psychological openness (eight items; e.g., “Psychological problems, like many things, tend to work out by themselves"); (2) Help-seeking propensity (eight items; e.g., “If I were experiencing a serious psychological problem, I would be confident that I could find relief in psychotherapy"); and (3) Indifference to stigma (eight items; e.g., “I would feel uneasy going to a professional because of what some people would think."). In the present study, only the last subscale was used. The total score of this subscale was calculated using the means of the scores obtained in the items that compose it, and higher scores reflected higher levels of stigma associated with the treatment of mental health problems. In our sample, the Cronbach’s alpha of this dimension was 0.83.

The intention to seek professional help was measured with the first part of the Portuguese version of the General Help-Seeking Questionnaire (GHSQ) [22,71]. This questionnaire evaluates the individual’s intention to request help from different sources (e.g., formal or informal) to deal with a mental health problem on a 7-point Likert scale, from 1 (Extremely unlikely) to 7 (Extremely likely). In this study, we only used the subscale intention to seek help through formal sources, whose score was obtained using the mean scores of the items “mental health professional” and “family physician”. Higher scores indicated greater intentions to seek help through these types of sources. For the present study, Cronbach’s alpha was 0.59.

In order to evaluate the intention to recommend help-seeking for mood and anxiety disorders in the postpartum period, we used the Questionnaire of Intentions to Recommend Professional Help-Seeking [29]. This questionnaire was constructed for Branquinho, Canavarro, and Fonseca’s [29] study, adapted from the questionnaire developed by Sprenger, Mettler, and Osma [72], based on Ajzen’s theory of planned behavior [64] to evaluate the general population’s intention to recommend professional help-seeking, in the context of postpartum depression. In the present study, the items were adjusted in order to evaluate men’s intentions to recommend professional help-seeking to their partner, in the context of maternal mood and anxiety disorders in the postpartum period. The questionnaire consists of 11 items, with a 7-point Likert-type response scale (1—Strongly disagree to 7—Strongly agree). The following components of Ajzen’s theory of planned behavior [64] were included in the items: (1) Attitudes (e.g., "Mental health services (psychology or psychiatry consultations) are useful for the treatment of postpartum depression/anxiety"); (2) Perceived behavioral control (e.g., "I am confident that I could encourage my partner to seek professional help (psychology or psychiatry consultations) if she displayed symptoms of depression/anxiety in the postpartum period"); (3) Subjective norms (e.g., "If my partner has depression/postpartum anxiety, it won’t look good if I encourage her to seek professional help (psychology or psychiatry consultations)"); and (4) Intentions (e.g., "I would be available to help my partner seek information about the services available for mental health problems (psychology or psychiatry consultations)"). Regarding construct validity, the structure of the Portuguese version of this questionnaire is unidimensional (*χ*^2^ = 158.36, *p* < 0.001; CFI = 0.93; RMSEA = 0.071) [29]. The total score was calculated using the means of the items, and higher scores indicated a greater intention to recommend help-seeking, in the context of maternal mood and anxiety disorders in the postpartum period. In our sample, Cronbach’s alpha was 0.69.

### 2.3. Statistical Analyses

Statistical analyses were performed with IBM SPSS (version 22.0, IBM Corp, Armonk, New York, USA) and AMOS (version 26.0, IBM Corp, Chicago, USA). Descriptive statistics were calculated to characterize the sociodemographic and clinical aspects of the sample. Stepwise regressions were performed to ascertain if type of recruitment (online vs. in-person) is significantly associated with the study variables, and therefore should be included as a covariate in the subsequent analyses.

Concerning the first goal of the study, a univariate analysis of covariance (ANCOVA) was performed to ascertain differences in the intention to recommend professional help-seeking to a partner in the postpartum period as a function of parenthood (no children, pregnant or youngest child up to one year old, youngest child over one year old). Additionally, Pearson’s bivariate correlations were calculated to evaluate the relationships between the sociodemographic and clinical variables of the sample and the intention to recommend professional help-seeking to a partner in the postpartum period. To perform these analyses, the variables professional status and previous treatment-seeking were recoded into dummy variables. Pearson’s *r* values close to 0.10 were interpreted as small correlations, *r* values close to 0.30 as moderate correlations, and *r* values equal to or greater than 0.50 as large correlations [73]. 

Concerning the second goal of the study, Pearson’s bivariate correlation coefficients among the variables under study were first computed (preliminary analyses). To examine the direct and indirect effects of the three dimensions of gender-role conflict on the intention to recommend professional help-seeking (through experiential avoidance, stigma, and the intention to seek professional help), a path analysis model was built in AMOS, using the maximum likelihood estimation method. In this model, the effect of the type of recruitment (online/in-person) and of monthly income were controlled, because they were significantly associated with the variables under study. The adjustment of the model was ascertained by the following adjustment indices [74]: qui-square (good adjustment of the model if *p* >0.05), comparative fit index (CFI; good adjustment of the model if >0.95), and root mean square error of approximation (RMSEA; good adjustment of the model if <0.06). In order to test the significance of the indirect effects, bootstrap procedures were used with 2000 samples and considering a confidence interval of 95% (bias-corrected 95% CI). Specific indirect effects and their confidence intervals were estimated using an AMOS user-defined estimand. The existence of indirect effects is considered if the zero value is not included in the confidence interval.

## 3. Results

### 3.1. Sociodemographic and Clinical Correlates of Men’s Intentions to Recommend Professional Help-Seeking to their Partners with PMAD

In the sample, the intention to recommend professional help-seeking to a partner in case they present mood and anxiety disorders in the postpartum period had a total mean score of 5.68 (*SD* = 0.75), out of a maximum possible total score of 7 points.

Type of recruitment was associated with some of the study variables (experiential avoidance: *B* = 0.211, *t* = 3.142, *p* = 0.002; and stigma: *B* = 0.142, *t* = 2.084, *p* = 0.038); therefore it was included as a covariate in the subsequent analyses. 

The intention to recommend professional help-seeking to a partner was not different among men who were currently in the perinatal period (i.e., whose partner was pregnant or had children up to one year old; *M* = 5.74, *SD* = 0.80), among men with older children (i.e., youngest child was over one year old; *M* = 5.68, *SD* = 0.70), or men without children (*M* = 5.60, *SD* = 0.74, *F*(3, 210) = 0.77, *p* = 0.510). Pearson’s correlation coefficients between the intention to recommend professional help-seeking to a partner in the postpartum period and the sociodemographic and clinical variables of the sample are presented in Table 2. The intention to recommend professional help-seeking to a partner was only significantly and positively correlated with the monthly income (small correlation). Specifically, having a higher monthly income was associated with a greater intention to recommend professional help-seeking to a partner in the postpartum period.

### 3.2. Direct and Indirect Effects of Gender-Role Conflict on the Intention to Recommend Professional Help-Seeking to a Partner with Mood and Anxiety Disorders in the Postpartum Period, Through Experiential Avoidance, Stigma, and Intention to Seek Professional Help

The means, standard deviations, and Pearson’s bivariate correlation coefficients between the variables under study are presented in Table 3. We highlight some significant associations, namely that the intention to seek professional help correlated positively and moderately with the intention to recommend professional help-seeking, with a greater intention to seek help associated with a greater intention to recommend professional help-seeking. Additionally, the dimensions of success, power and competition, restrictive emotionality, conflicts between work and family relations (of gender-role conflict), stigma, and experiential avoidance were found to be significant, and negatively associated with the intention to recommend professional help-seeking. In this sense, higher levels of gender-role conflict, stigma, and experiential avoidance were associated with a lower intention to recommend professional help-seeking.

**Figure 2:**
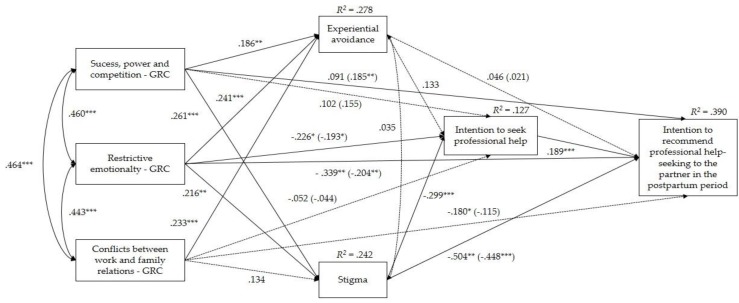
Mediation model with determinants of the intention to recommend professional help-seeking to a partner with mood and anxiety disorders in the postpartum period. The values represented in the lines correspond to standardized values. Those out of parentheses present the total effects, and those in parentheses, the direct effects, after the introduction of the mediating variables in the model. There was a significant positive association between experiential avoidance and type of recruitment (*B* = 0.188, *SE* = 0.212, *p* = 0.007). The remaining associations with covariates were not significant; * *p* < 0.05; ** *p* < 0.01; *** *p* < 0.001.

The model that explores the determinants of men’s intentions to recommend professional help-seeking to their partners in the postpartum period is represented in Figure 2. In this model, the type of recruitment and the monthly income were inserted as covariates, given the former’s significant association with stigma and experiential avoidance, and the latter’s significant association with the intention to recommend professional help-seeking. The model revealed a very good adjustment to the data (*χ*^2^(12) = 19.14, *p* = 0.085; CFI = 0.982; RMSEA = 0.053, *p* = 0.411, [90% CI = 0.000, 0.095]).

Table 4 presents the path coefficients of the final model. As shown in Figure 2 and on Table 4, the three dimensions of gender-role conflict (success, power, and competition; restrictive emotionality; conflicts between work and family relations) were significant and positively correlated with each other. Additionally, higher levels of the three dimensions of gender-role conflict were significantly associated with higher levels of experiential avoidance. Higher levels of the dimensions of success, power, and competition and restrictive emotionality were significantly associated with higher levels of stigma. Also, higher levels of stigma were significantly associated with a lower intention to seek professional help, but the same did not apply to experiential avoidance. Finally, a greater intention to seek professional help was significantly associated with a greater intention to recommend professional help-seeking to a partner in the postpartum period.

With regards to the total, direct, and indirect effects of gender-role conflict on the intention to seek professional help, it was found that the total and direct effects of the dimension success, power, and competition on the intention to seek professional help were not significant, and this relationship also did not occur indirectly (−0.101, [−0.254, 0.022]). Regarding the total and direct effects of the dimension restrictive emotionality on the intention to seek professional help, both were significant, indicating that higher levels of restrictive emotionality were associated with a lower intention to seek professional help. This relationship did not occur indirectly (−0.047, [−0.148, 0.053]). The total, direct, and indirect effects (−0.011, [−0.085, 0.058]) of the dimension conflicts between work and family relations on the intention to seek professional help were not significant.

Regarding the total, direct, and indirect effects of gender-role conflict on the intention to recommend professional help-seeking, the direct effect of the dimension success, power, and competition on the intention to recommend professional help-seeking was significant, revealing that higher levels of success, power, and competition have been associated with a greater intention to recommend professional help-seeking to a partner with mood and anxiety disorders in the postpartum period. Still within this relationship, an indirect effect was verified (−0.076, [−0.147, −0.010]), which occurs through the variables stigma (−0.106, [−0.168, −0.056]), intention to seek professional help (0.027, [0.003, 0.065]), and the sequence stigma-intention to seek professional help (−0.013, [−0.034, −0.005]), but not through experiential avoidance or the sequence experiential avoidance-intention to seek professional help. This indicates that higher levels of success, power, and competition are reflected in higher levels of stigma, which in turn leads to a lower intention to seek professional help and, consequently, to recommend it.

The total and direct effects of the dimension restrictive emotionality on the intention to recommend professional help-seeking were significant, and an indirect effect was verified too (−0.076, [−0.131, −0.029]), through the variables stigma (−0.066, [−0.118, −0.028]), intention to seek professional help (−0.013, [−0.028, −0.005]), and the sequence stigma-intention to seek professional help (−0.008, [−0.021, −0.002]), but not through the variable experiential avoidance or the sequence experiential avoidance-intention to seek professional help. Thus, higher levels of restrictive emotionality were associated with higher levels of stigma, which led to a lower intention to seek professional help and, consequently, to recommend this type of help. 

The total effect of the dimension conflicts between work and family relations on the intention to recommend professional help-seeking was significant; however, the direct and indirect (−0.041, [−0.100, 0.006]) effects proved to be nonsignificant.

## 4. Discussion

The present study is innovative in characterizing men’s intentions to recommend professional help-seeking to a partner, in cases where they display PMAD. Additionally, it contributes to the knowledge of the factors and mechanisms influencing these intentions. As main results, our study found that: (1) Men of the Portuguese population have a high intention to recommend professional help-seeking to their partners, in the presence of PMAD; (2) Men with a higher monthly income have a greater intention to recommend professional help-seeking to their partners in the postpartum period; (3) High levels of restrictive emotionality and conflicts between work and family relations, and reduced levels of success, power, and competition are directly associated with lower intentions to recommend professional help-seeking to a partner in the postpartum period; and (4) The effect of the dimensions success, power, and competition and restrictive emotionality on the intention to recommend professional help-seeking to a partner in the postpartum period seems to occur indirectly and sequentially, through the occurrence of higher levels of stigma, and consequently, lower intentions to seek professional help, which in turn translates into a lower intention to recommend professional help-seeking to their partners. 

Our results suggest that the sample under study presents a high intention to recommend professional help-seeking to their partners in the case of PMAD. Though the effect of social desirability cannot be excluded, this data is congruent with a systematic review and recent meta-analysis that demonstrated that, over the last two decades, the general population has shown more positive attitudes towards professional help-seeking (psychiatrists and psychologists), along with an increase in the intention to recommend professional help-seeking for psychiatric problems [28]. Moreover, the pattern of intentions to recommend help-seeking to a partner in the postpartum period was similar for men who are currently in the perinatal period, men with older children, and childless men. Our results suggest that even without the experience of parenthood, men are able to position themselves in a hypothetical situation in which their partners are experiencing emotional difficulties in the postpartum period, or at least that they can position themselves hypothetically in a situation in which their partners may experience emotional difficulties (albeit unrelated to the postpartum period). These results point to the justification of the design of universal awareness campaigns for the male population (currently in a relationship with a partner within the reproductive age), as opposed to campaigns only directed at men whose partners are in the perinatal period. Finally, men with higher monthly incomes have shown a greater intention to recommend help. This result seems to be understandable, since in the literature, practical/structural barriers are identified in relation to the intention and decision to seek professional help [9,23,75]. Thus, a higher monthly income could minimize this obstacle and allow access to more alternatives regarding the use of mental health services (e.g., recourse to the private sector), thus increasing men’s intentions to recommend professional help-seeking to their partners.

Concerning the relationship between gender-role conflict and the intention to recommend professional help-seeking, the three dimensions of this conflict were associated with the intention to recommend help-seeking. However, in the case of success, power, and competition (direct effect), the direction of the relationship was not expected [37,44,46], since high levels of this dimension were associated with a greater intention to recommend professional help-seeking, contrary to what was observed for the remaining dimensions, in which higher levels of restrictive emotionality (total and direct effects) and conflicts between work and family relations (total effect) resulted in a lower intention to recommend professional help-seeking. To the best of our knowledge, the only study that focused on the influence of gender-role conflict on the intention to recommend professional help-seeking to others only included the dimension of restrictive emotionality in its analysis, out of the dimensions in our study [47]. One possible explanation for the results found for the dimension success, power, and completion in our study may be the fact that this dimension may not absolutely reflect the negative consequences resulting from male gender roles [76]. In fact, O’Neil [40] argued that this dimension is essentially a measure of ideology and masculine norms, assessing the level of conflict indirectly, through attitudes towards achieving success via power and competition. Additionally, in the Portuguese version of the scale, one of the items of this dimension seems to convey a protective characteristic towards others ("I often feel that I have to take care of those around me"). As such, the dimension of success, power, and competition may also have included a facet of care and protection, which understandably may have increased the intention to recommend professional help-seeking to a partner. Moreover, this explanation is in line with the results of one study that showed a positive relationship between success, power, and competition and the intention to seek professional help for substance abuse disorders, in a sample of men from Costa Rica [77]. It is possible that the participants in our sample may not have interpreted the act of recommending professional help-seeking as admitting a failure capable of threatening their perception of success and power [37], or of generating a feeling of failure as a spouse [48], but as a way of demonstrating power through taking control of the situation [77] by expressing the intention to recommend help-seeking to their partner. Considering the divergence of the results found in the literature, further studies are needed to clarify this relationship.

Although, as previously mentioned, the dimension of success, power, and competition is directly associated with a greater intention to recommend professional help-seeking, this influence also occurs indirectly, and in an opposite direction. Specifically, higher levels of success, power, and competition are associated with higher levels of stigma, which in turn leads to a lower intention to seek, and consequently, recommend help to a partner. This can happen due to the suppressor effect that (in this context) the stigma variable seems to exert. After its introduction into the model, the relationship between this dimension of gender-role conflict and the intention to recommend professional help-seeking intensifies (i.e., the direct effect was observed to be greater than the total effect). Thus, in the presence of high levels of stigma, the dimension of success, power, and competition seems to activate concerns related to what others might think about the individual or their partner’s behavior of seeking help, leading to a lower intention to seek professional help and to recommend it. This result suggests a complex relationship between these variables, so future studies should investigate other mechanisms that may influence this process.

In line with our results, there is evidence that higher levels of restrictive emotionality are associated with lower male intentions to seek [37,44,46] and recommend professional help [47]. By restricting the expression of emotions, men may find it difficult to identify and discuss them [47], making it difficult to express concern about the depressive or anxious symptomatology of their partner, which, therefore, may result in the nonrecommendation of professional help-seeking. Contrary to the results found by Vogel and colleagues [47], our study showed that the relationship between restrictive emotionality and the intention to recommend professional help-seeking is mediated by the sequence stigma-intention to seek professional help (and for each of these variables individually). This situation may be due to the fact that the stigma variable is defined and evaluated differently in the study conducted by these authors, since they focused on the stigma directed at individuals who have experienced mental health problems (and not the stigma related to help-seeking). In our study, the stigma related to help-seeking reflects a state of discomfort of the individual towards what others may think when they find out that they sought professional help [55]. Although not restricted to the individual, since the discomfort felt can be generalized to what others might think about their partner, the variable stigma seems to assume a more intrapersonal character here. Thus, it is understandable that a greater degree of conflict associated with the difficulty of expressing emotions results in higher levels of stigma (i.e., in a perception that those seeking and receiving treatment for mental health problems are not socially accepted) [54], which in turn results in a lower intention to seek professional help, and consequently, to recommend it to a partner.

Compared with the other dimensions of gender-role conflict studied, in the relationship between the dimension conflicts between work and family relations and the intention to recommend professional help-seeking, only the total effect was significant. The literature shows that the experience of conflicts between work and family is associated with a reduction of health-related behaviors [78,79] and with less intention to pursue a healthy lifestyle [80]. Furthermore, this type of conflict is associated with a lower quality of romantic relationships [81] and parental conflicts [82], where men might present more hostile interactions with their partners [83] and assume a minor level of involvement in family life [84]. Thus, the nonprioritization of health aspects, allied to relational conflicts, may lead to a lower intention to recommend help-seeking to their partners. Future studies should take into account the aspects mentioned above, due to their possible mediator role in the relationship between the dimension of conflicts between work and family relations and the intention to recommend professional help-seeking to a partner. 

Finally, in our study, the relationship between the different dimensions of gender-role conflict and the intention to recommend professional help-seeking was not mediated by the levels of experiential avoidance. Although, as expected, high levels of the dimensions of gender-role conflict are associated with high levels of experiential avoidance [85], this last variable was positively, but not significantly, associated with the two intentions studied. These results are unexpected, given that the use of experiential avoidance strategies is likely to lead to an inability to take the necessary measures to deal with negative internal experiences [50,51]. It is possible, however, that there are other variables not contemplated in our model that might explain the results obtained. For example, studies show that high mental health literacy and a high level of knowledge about postpartum depression are associated with more positive attitudes towards help-seeking and with a greater intention to recommend professional help-seeking, respectively [29,86]. Additionally, the prior use of mental health services is related to a higher probability of recommending help-seeking for anxious and depressive symptomatology [87]. Thus, it is possible that these types of variables may play a relevant role in explaining the relationship between experiential avoidance and the intention to seek and to recommend professional help, and should be considered in future studies.

Despite the innovative contributions of our study, the interpretation of our results should be cautious, considering the existence of some limitations. Firstly, this is a cross-sectional study, and therefore, it is not possible to establish causality relationships between the study variables. Future studies should seek to replicate the results through a longitudinal methodology, analyzing, if possible, the behavior of professional help-seeking (and not just the intention). 

Secondly, our results may be conditioned by the fact that most of the sample was recruited online, and it is possible that only men who were more interested in and conscious of the topic of mood and anxiety disorders in the postpartum period, and of professional help-seeking, might have completed the questionnaire. Thus, the fact that a large part of the sample has been self-selected may contribute to explaining the high levels of the intention to recommend professional help-seeking found in our sample. Moreover, the sample of our study may not be representative of all men in the Portuguese population who are currently in a relationship with a partner within the reproductive age, since most of the participants were married or cohabiting with their partners and with a monthly income between 1001€ and 2000€. Accordingly, future studies should use a larger and sociodemographically more diversified sample. It should also be noted that the results may have been affected by the social desirability and/or the nature of the high levels of gender-role conflict, which could condition the availability of men to demonstrate attitudes and behaviors with feminine connotations (e.g., seeking for help), thus compromising the data obtained in the self-reported questionnaires. Future studies would benefit from the inclusion of a questionnaire to assess social desirability, in order to control the effect of this variable on the results. Moreover, data concerning the participant’s ethnic background was not collected in the present study. Future studies should assess the role of ethnic background and of cultural diversity in the men’s intentions to recommend professional help-seeking to their partners with PMAD.

Thirdly, it is important to mention the low internal consistency of the scales that assessed the intention to recommend professional help-seeking for maternal mood and anxiety disorders in the postpartum period (*α* = 0.69, which is slightly below the acceptable threshold), and particularly, the intention to seek professional help (*α* = 0.59; although these levels of internal consistency can be explained by the fact that Cronbach’s alpha is sensitive to the number of items and this scale has only two items), which could also have compromised the interpretation of the results.

In addition to clarifying the exploratory relationships found in the present study, it would be important for future studies to analyze other specific dimensions of stigma in the relationship between gender-role conflict and the intention to recommend professional help-seeking to a partner, in the presence of PMAD. Specifically, given the focus of the male gender-role in the need for power and status, future studies should investigate the mediator role of the variable that Goffman [88] designated as “courtesy stigma”. This is a stigma that, while affecting a person with mental health problems, also affects those who are closely associated with them [89,90]. Thus, men with a greater degree of gender-role conflict may have a lower intention to recommend professional help-seeking to their partners, considering the fear of also being a target of rejection, prejudice, and discrimination as a member of the couple, thus decreasing their sense of success and power.

## 5. Conclusions

To the best of our knowledge, this was the first study which explored men’s intentions to recommend professional help-seeking in cases where their partners presented PMAD, and the factors that could influence it. In our study, we found evidence for the influence of the three dimensions of gender-role conflict on the intention to recommend professional help-seeking. The dimension restrictive emotionality seems to stand out, by negatively influencing the intention to recommend professional help-seeking before and after the introduction of mediating variables in the model, and, indirectly, through the sequence stigma-intention to seek professional help. Moreover, higher levels of success, power, and competition proved to be indirectly associated with the intention to recommend help-seeking, through the sequence stigma-intention to seek professional help. Considering that only the total effect of the dimension conflicts between work and family relations on the intention to recommend professional help-seeking was significant, future research should explore this relationship, by examining the role of other mediator variables. 

Given the decisive influence that men have in their partners’ process of seeking professional help in the postpartum period, it is extremely important to include men who are currently in a relationship with a partner within the reproductive age in universal education and awareness-raising campaigns [11,21] that can promote an increase in men’s intentions to recommend professional help-seeking to their partners in cases of necessity. These should be centered on the theme of mood and anxiety disorders (in the postpartum period), addressing their symptoms and the negative impact they have on women and families, and should clarify the importance of men’s encouraging roles in their partner’s process of seeking professional help [21]. However, it is important that these campaigns directed at men also include, directly or indirectly, aspects related to the expression of emotions and concern for others, and the balance between family and employment/studies, which, according to our results, could promote a lower negative influence of gender-role conflict on the intention to recommend professional help to a partner. Moreover, awareness-raising and education campaigns should also aim to reduce the stigma of seeking professional help, and to promote professional help-seeking behavior, for example, by discussing its advantages [54] and by challenging the notion of help-seeking as a sign of weakness [91], which results in social nonacceptance [54]. This should enhance the intention to seek professional help and to recommend it to their partner, to address her difficulties either during the postpartum period or in other life periods.

## Figures and Tables

**Table 1:** Sociodemographic, clinical and other characteristics related to the partner (*N* = 214).

Sociodemographic Characteristics		
	M (SD)	Min–Max
Age	32.61 years (6.18)	18–51
	*n*	%
Education		
Basic education	32	14.9
Secondary education	88	41.1
Bachelor’s degree	65	30.4
Master’s degree	25	11.7
Doctoral degree	4	1.9
Professional status		
Employed	192	91
Unemployed	3	1.4
Student	16	7.6
Retired	0	0
Other	2	0.9
Monthly income		
≤500€	4	1.9
500€–1000€	39	1.2
1001€–2000€	118	55.1
2001€–3500€	46	21.5
3501€–4500€	3	1.4
≥4500€	4	1.9
Residence		
Rural	78	36.8
Urban	134	63.2
Relationship status		
Married/cohabitating	168	79.2
In a relationship (without cohabitation)	44	20.8
Relationship duration	9.08 years (5.83)	0.4–39
Parental Status		
No	62	29
Yes, in the perinatal period (partner currently pregnant or youngest child below one year old)	80	37.4
Yes, youngest child over one year old	72	33.6
Clinical Characteristics		
History of psychological problems		
Yes	36	16.8
No	178	83.2
Previous treatment-seeking		
No	163	76.2
Yes, psychiatric	9	4.2
Yes, psychological	29	13.6
Yes, both (psychological and psychiatric)	13	6.1
Current psychological problems		
Yes	20	9.3
No	194	90.7
Clinical Characteristics of the Partner		
History or current presence of psychological problems		
Yes	81	3.9
No	133	62.1
Previous treatment-seeking		
No	145	67.8
Yes, psychiatric	10	4.7
Yes, psychological	47	22
Yes, both (psychological and psychiatric)	12	5.6
Presence of psychological problems in the perinatal period		
Yes	42	29.8
No	99	70.2
Treatment seeking in the perinatal period		
No	126	89.4
Yes, psychiatric	5	3.5
Yes, psychological	10	7.1
Yes, both (psychological and psychiatric)	0	0

**Table 2:** Pearson’s correlation between the intention to recommend professional help-seeking to a partner with mood and anxiety disorders in the postpartum period and the sociodemographic and clinical variables.

Sociodemographic and Clinical Variables	Intention to Recommend Help-Seeking
Age	0.003
Education	−0.058
Parental status	0.043
Monthly income	0.137 *
Residence	0.020
Relationship status	0.036
Professional situation	−0.026
History of psychological problems	0.037
Previous treatment-seeking	0.011
Current psychological problems	−0.110

Note. Residence (1 = rural; 0 = urban); Professional status (1 = employed; 0 = unemployed, student, retired, or other); History of psychological problems (1 = yes; 0 = no); Previous treatment-seeking (1 = yes; 0 = no); Current psychological problems (1 = yes; 0 = no); * *p* < 0.05.

**Table 3:** Descriptives and Pearson bivariate correlations between the study variables.

Study Variables	M	SD	1.	2.	3.	4.	5.	6.
1. SPC_GRC	3.48	0.82						
2. RE_GRC	3.24	1.09	0.460 ***					
3. CBWFR_GRC	3.76	1.23	0.464 ***	0.443 ***				
4. Exp_Avoid	19.79	8.31	0.421 ***	0.438 ***	0.430 ***			
5. Stigma	0.83	0.74	0.431 ***	0.400 ***	0.353 ***	0.291 ***		
6. Intent_Seek	4.61	1.57	−0.027	−0.203 **	−0.105	0.009	−0.287 ***	
7. Intent_Recom	5.68	0.75	−0.152*	−0.390 ***	−0.291 ***	−0.183 **	−0.542 ***	0.367 ***

Note. Exp_Avoid: Experiencial avoidance; SPC_GRC: Success, power, and competition (gender-role conflict); RE_GRC: Restrictive emotionality (gender-role conflict); CBWFR_GRC: Conflicts between work and family relations (gender-role conflict); Intent_Seek: Intention to seek professional help; Intent_Recom: Intention to recommend professional help-seeking; * *p* < 0.05; ** *p* < 0.01; *** *p* < 0.001.

**Table 4:** Path coefficients of the final model (direct effects).

Direct Effects	*B*	*p*
Success, power, and competition (GRC) -> Experiential avoidance	0.186	0.006
Restrictive emotionality (GRC) -> Experiential avoidance	0.241	<0.001
Conflicts between work and family (GRC) -> Experiential avoidance	0.233	<0.001
Success, power, and competition (GRC) -> Stigma	0.261	<0.001
Restrictive emotionality (GRC) -> Stigma	0.216	0.002
Conflicts between work and family (GRC) -> Stigma	0.134	0.056
Success, power, and competition (GRC) -> Intention to seek professional help	0.155	0.052
Restrictive emotionality (GRC) -> Intention to seek professional help	−0.193	0.014
Conflicts between work and family (GRC) -> Intention to seek prof. help	−0.044	0.578
Success, power, and competition (GRC) -> Intention to recommend help	0.185	0.006
Restrictive emotionality (GRC) -> Intention to recommend help	−0.204	0.002
Conflicts between work and family (GRC) -> Intention to recommend help	−0.115	0.076
Experiential avoidance -> Intention to seek professional help	0.133	0.078
Stigma -> Intention to seek professional help	−0.299	<0.001
Experiential avoidance -> Intention to recommend help	0.021	0.739
Stigma -> Intention to recommend help	−0.448	<0.001
Intention to seek professional help -> Intention to recommend help	0.189	<0.001
